# EEG Functional Connectivity Analysis for the Study of the Brain Maturation in the First Year of Life

**DOI:** 10.3390/s24154979

**Published:** 2024-08-01

**Authors:** Anna Falivene, Chiara Cantiani, Chiara Dondena, Elena Maria Riboldi, Valentina Riva, Caterina Piazza

**Affiliations:** Scientific Institute IRCCS E. Medea, 23842 Bosisio Parini, Italy; anna.falivene@lanostrafamiglia.it (A.F.); chiara.dondena@lanostrafamiglia.it (C.D.); elenamaria.riboldi@lanostrafamiglia.it (E.M.R.); valentina.riva@lanostrafamiglia.it (V.R.); caterina.piazza@lanostrafamiglia.it (C.P.)

**Keywords:** functional connectivity, baseline EEG, brain development, infants

## Abstract

Brain networks are hypothesized to undergo significant changes over development, particularly during infancy. Thus, the aim of this study is to evaluate brain maturation in the first year of life in terms of electrophysiological (EEG) functional connectivity (FC). Whole-brain FC metrics (i.e., magnitude-squared coherence, phase lag index, and parameters derived from graph theory) were extracted, for multiple frequency bands, from baseline EEG data recorded from 146 typically developing infants at 6 (T6) and 12 (T12) months of age. Generalized linear mixed models were used to test for significant differences in the computed metrics considering time point and sex as fixed effects. Correlational analyses were performed to ascertain the potential relationship between FC and subjects’ cognitive and language level, assessed with the Bayley-III scale at 24 (T24) months of age. The results obtained highlighted an increased FC, for all the analyzed frequency bands, at T12 with respect to T6. Correlational analyses yielded evidence of the relationship between FC metrics at T12 and cognition. Despite some limitations, our study represents one of the first attempts to evaluate brain network evolution during the first year of life while accounting for correspondence between functional maturation and cognitive improvement.

## 1. Introduction

Connectivity analysis offers insight into brain topology and interactions, as well as how these connections may change during development and growth. The first year of life is in fact a critical phase of postnatal brain development, since in this period cerebral changes are particularly rapid, intense and crucial for the baby’s acquisition of first cognitive, linguistic and motor skills [1,2].

Studies on connectivity aim to map and describe communication between different areas of the brain [3]. In this context, neural connectivity analysis is classified into three different categories [4]: structural, functional and effective connectivity. 

Structural connectivity analysis is the study of the anatomical connections between fibers in different areas of the brain [5]. For this purpose, imaging techniques, such as Magnetic Resonance Imaging (MRI) and particularly Diffusion Tensor Imaging (DTI), are commonly applied thanks to their high spatial resolution [3,4]. 

Functional connectivity (FC) identifies dynamic connections, interaction and statistical dependence among neural elements [3]. In this field, functional MRI (fMRI), electroencephalography (EEG), or magnetoencephalography (MEG) studies [6] are typically adopted [7]. Specifically, when dealing with EEG signals, commonly used FC measures include the Pearson product moment correlation coefficient [5], which evaluates the degree of mathematical association between two different amplitudes in the time domain; and the coherence [4], which measures the strength of signal connection in both time and frequency domains. However, it is important to note that signal coherence evaluation can be influenced by the problem of volume conduction leading to spurious correlations [8]. An alternative approach, introduced by Stam and colleagues [8], is the computation of the phase lag index (PLI), a measure of phase synchronization that reflects the strength of the coupling of two signals. The PLI is considered to be less sensitive to the influence of volume conduction since its computation relies upon the hypothesis that the existence of a consistent and nonzero phase lag between two timeseries cannot be explained by volume conduction from a single strong source. Other parameters, based on the phase coupling between signals, can be used to assess FC; for a review, see [9]. 

Furthermore, graph theory has often been applied both in structural and functional connectivity analysis, offering a more comprehensive understanding of the networks’ architecture [10], which are represented as trees composed by a sets of nodes (i.e., brain regions), and links between them (i.e., anatomical or functional connections).

Finally, effective connectivity aims at classifying causal interactions between neural elements [3]. While functional connectivity identifies whether brain areas are interacting, effective connectivity allows for the assessment of the direction in which the information flows [5]. One of the most used metrics is the Granger causality measure, which determines the direction of the connections, predicting whether and to what extent previous EEG activity of one channel (i.e., over one brain area) may influence the future activity of another EEG channel [11]. 

Focusing on the developmental literature, to date, many studies aimed at analyzing the maturational process underlying brain networks by assessing structural changes or using mainly fMRI technique [2,12,13]. In terms of structural connectivity, the literature has demonstrated that during development, the brain undergoes a significant reorganization through synaptogenesis and pruning, from a relatively randomized configuration to a more organized and stable one [2,14]. These modifications lead to greater integration, robustness, and efficiency of neural circuits during the first two years [14]. As for functional connectivity, Gao and collaborators analyzed brain networks of 147 children between the first 3 weeks and 2 years of life using resting-state fMRI, finding a notable improvement in brain wiring efficiency during the first year that remained more stable at 2 years of age [15]. Lemaître and colleagues analyzed 52 infants from 3 to 12 months of age with MRI in resting condition to assess the dynamics of local functional maturation. The obtained results showed marked regional differences in the brain maturation at the cortical level (early maturation in the primary sensorimotor cortices; temporal and prefrontal region presented increasing maturation in the first year; late maturation in the posterior superior temporal cortices) [1]. 

Despite a relatively lower spatial information, EEG seems to be more convenient with respect to fMRI for the study of early brain development thanks to a superior temporal resolution and higher suitability for very young age groups [13]. So far, only a few studies assessed functional brain networks in typically developing infants and children by means of EEG systems. Tóth and her team analyzed EEG recordings of 139 healthy full-term infants in their first week of life, during quiet sleep. PLI and MST metrics were derived for the acquired data and compared with the ones of a referential random network, suggesting the presence of an early organization and hierarchical architecture in all frequency bands [16]. Boersma and collaborators recorded EEG signals from 227 children at both 5 and 7 years of age in a resting condition with eyes closed. Graph theory was applied, revealing changes from 5-to-7-year-brain functional networks towards a more structured organization [17]. Bathelt applied source reconstruction with age-matched templates to task-free high-density EEG recordings in typically developing children between 2 and 5 years of age. Functional connectivity was explored with graph analysis, reporting network development towards more closely integrated networks [18]. To the best of our knowledge, no previous studies have used EEG FC to investigate typical brain development in the first year of life. In this context, baseline EEG represents an ideal condition, since it allows the investigation of the brain organization at the level of the individual while no assigned task is being performed. Baseline EEG is widely used across the lifespan. Specifically, during infancy, it consists in the recording of the EEG signal during short periods of quiet wakefulness while the infants watch a minimally arousing video clip, abstract shapes moving on a screen, colorful balls spinning, or a live person blowing bubbles or manipulating an object [19].

When dealing with maturational analysis, it is also important to take into account how sex may affect brain connectivity in order to assess associated differences, as largely demonstrated by the literature evidence [20]. Specifically, the literature is in favor of higher FC in females than in males both during adulthood and developmental age. Tomasi et al. investigated sex differences in resting-state functional brain connectivity among 336 women and 225 men, ranging in age from 18 to 30 years by means of fMRI recordings, finding higher local functional connectivity in women with respect to males [21]. Wang and collaborators analyzed functional connectivity in 104 4-month-old infants during sleep assessed with functional near-infrared spectroscopy, to identify potential differences between male and female infants. Specifically, Pearson correlation adjacency matrices were built finding a general higher connectivity in females [22]. However, more research is necessary to better explore the association between gender and FC, especially during brain development. Moreover, also in this field, the presence of EEG research is relatively scarce [20].

Finally, previous research has started to investigate the organization of functional brain systems during development and how they adapt to the maturational process of cognitive functions. For instance, functional connectivity was explored in relation to language abilities by Perani et al. by means of resting-state fMRI in 15 healthy 2-day-old infants [23]. The study revealed the presence of immature language networks at birth, which were characterized by strong interhemispheric connections, with the assumption that the progressive maturation of intra-hemispheric functional connectivity would be established with language exposure and brain development. Bruchhage and colleagues investigated the development of FC during infancy and childhood and the relationship with the emergence of cognitive abilities in 196 typically developing children between 3 months and 6 years of age, using fMRI recordings during sleep. Specifically, the correlation analysis on the FC with raw scores of the Mullen Scales of Early Learning for visual reception, fine and gross motor, receptive and expressive language (corrected for child age) demonstrated increased functional connectivity with networks involved in broader higher cognitive functions [24].

As for EEG analysis, previous literature has focused mostly on the role of gamma activity, given the link between cortical activity in the gamma frequency range and various high-cognitive processes. In particular, increased power within the gamma frequency range in response to selected stimuli has been linked to different psychological, cognitive and perceptual processes such as object recognition (e.g., [25]), perceptual learning (e.g., [26]), attention (e.g., [27]), memory (e.g., [28]) and language (e.g., [29,30,31]). Moreover, in the work conducted by Gou and colleagues, frontal gamma power measured longitudinally in 40 infants, with no diagnosed language or learning disabilities, at 16, 24 and 36 months of age, was found to predict language outcomes at 4 and 5 years, thus supporting the role of early gamma activity on language development [32]. A similar result was found by Brito et al., who recorded baseline parietal gamma power in 66 typically developing newborns, finding a positive correlation with linguistic abilities at 15 months of age [33]. Furthermore, in a longitudinal study of 84 infants [34], a predictive role of gamma oscillatory activity at 6 months on later language acquisition at 24 months was found.

Although all the discussed topics have been covered previously, the literature lacks baseline EEG studies focused mainly on the first year of life, also combining analysis on functional connectivity development and its link to cognition.

Therefore, the main aim of the current study was to assess network developmental changes in infants in the second half of the first year of life, which is the most critical for brain development, by means of baseline EEG FC, accounting for sex-related effects as well. Furthermore, the association with later cognitive and language outcomes was explored trough correlational analysis between FC features extracted in the gamma frequency band and the Bayley scale scores, collected at 2 years of age. In the present work, we focused only on FC metrics, which we think represents the more suitable connectivity measures to assess both developmental changes and associations with the cognitive function development.

## 2. Materials and Methods

### 2.1. Participants

The study sample was recruited within a larger ongoing longitudinal project [34,35]. In the present study, the sample consisted of 146 typically developing infants (see Table 1), including 132 6-month-olds (T6) and 61 12-month-olds (T12), for EEG recording sessions. Out of the 146 subjects, signals from 47 infants were recorded at both ages, so that a total of 193 EEG signals were collected. 

Inclusion criteria were the following:

1. Gestational age ≥ 36 weeks;
2. Bayley Cognitive Composite Score ≥ 85 [37] or Griffiths developmental quotient ≥ 85 [38], both assessed at 6 months of age;
3. No certified diagnosis of intellectual deficiency or neurodevelopmental disorders;
4. At least one native Italian-speaking parent.

**Figure 1:**
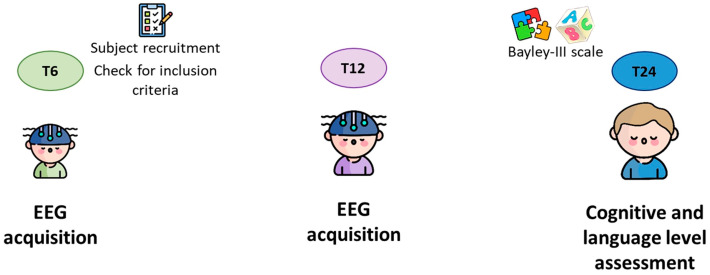
Schematic illustration of the data collection procedure of the study.

Before inclusion in the protocol, all parents were informed about the methodology and duration of the study (Figure 1) and gave their written informed consent to participate. The study was conducted in accordance with the Declaration of Helsinki and approved by the Ethics Committee of the Scientific Institute IRCCS E. Medea (Bosisio Parini, Italy).

### 2.2. EEG Data Acquisition and Pre-Processing

The EEG acquisition protocol and pre-processing were the same described in [35]. 

Baseline EEG was acquired for four minutes, during which infants were seated on their parents’ laps in a sound-attenuated and electrically shielded room, and a research assistant blew bubbles to keep them quiet.

A dense-array EGI system (Geodesic EEG System (GES) 300 or 400, Electric Geodesic, In., Eugene, OR, USA) equipped with 60/64-electrode or 128-electrode caps (HydroCel Geodesic Sensor net) was used. Data were referenced to the vertex, recorded with a sample frequency of 250 Hz or 1000 Hz and bandpass-filtered between 0.1 and 100 Hz. 

**Figure 2:**
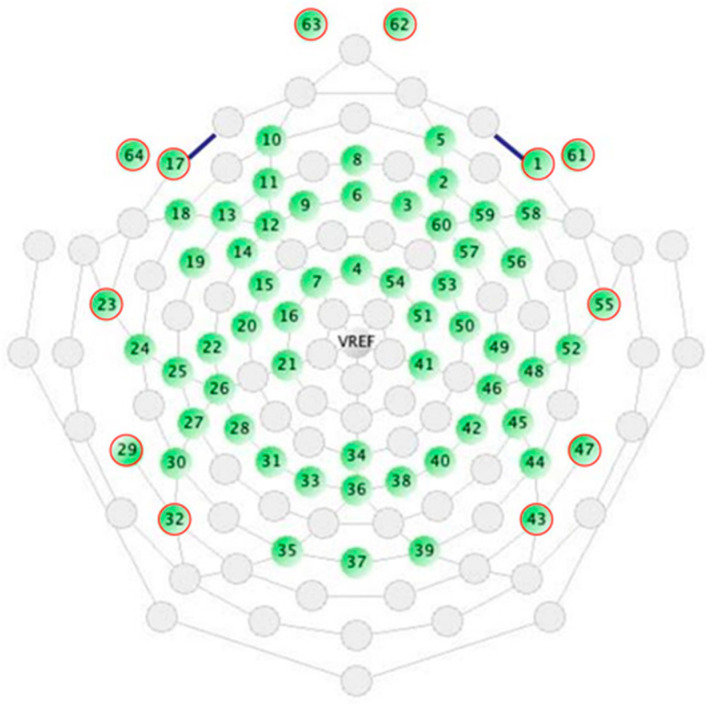
EEG channels reference configuration [44]. Red-circled channels are the ones removed from the described processing pipeline.

Pre-processing of raw data was performed with the open-source EEGLAB signal processing environment and custom MATLAB scripts (The Mathworks, Natick, MA, USA): first, data acquired with a sample frequency of 1000 Hz were downsampled at 250 Hz. The 60-electrode cap was taken as reference, since 94.5% of recruited subjects’ data were collected with this cap. Therefore, the signals recorded in the channels exceeding the ones available in the reference cap were excluded from further analysis (i.e., ocular channels 61, 62, 63 and 64 (Figure 2); and 128-electrode cap additional channels). Continuous EEG data were then filtered with a 1 Hz high-pass and a 47 Hz low-pass FIR filter. Bad channels and noisy portions of data were identified and removed exploiting the clean_rawdata EEGLAB plugin according to the criteria described in [35]; only data with at least 1 min of good signal and no more than 15 channels identified as bad were further analyzed. Bad channels were interpolated with a spherical spline, and the average re-reference was performed. 

Independent component analysis (ICA) was applied by means of the RUNICA Infomax algorithm [39]. The independent components (ICs) accounting for artifactual activities were then identified using the ICLabel plugin [40] and removed [35] (number of removed ICs: M = 12.1, SD = 3.7). Additionally, 8 of the outermost channels (channels 1, 17, 23, 29, 32, 43, 47 and 55 of the 60-channel cap) were discarded from further analysis, as they could be the most affected by artifacts and presented high interpolation rates (M = 26.1, SD = 6.1), resulting in a total of 52 channels (Figure 2). Finally, the EEG signals were segmented into 2-s epochs, and the first 15 artifact-free epochs were selected for each subject. This ensured at least 30 s of cleaned signal for all subjects. We based our choice according to previous and similar studies (e.g., [41,42,43]), who performed functional connectivity analysis on 5-s (or less) epochs of EEG signals.

### 2.3. Functional Connectivity Analysis

The focus of the current study is on the functional connectivity analysis. 

The magnitude squared coherence (MSC) was computed, as in (1), by normalizing the cross-power spectral density function ($S_{xy}$) of *x* and *y* EEG channels with their respective auto-power spectral density functions ($S_{xx}$ and $S_{yy}$), which were calculated using Welch’s averaged modified periodogram method [4].

(1) $$MSC\left(f\right)=\frac{{\left|<S_{xy}\left(f\right)>\right|}^{2}}{\left|<S_{xx}\left(f\right)>||<S_{yy}\left(f\right)>\right|}$$

The use of < > symbols indicates window averaging, whereas || refers to absolute value computation.

The estimated MSC for a given frequency *f* ranges between 0 (i.e., no signal coupling between pairs of channels) and 1 (i.e., maximum linear inter-dependence). A squared 52 × 52 MSC matrix was then constructed, from which the mean value (Mean MSC) was computed as a whole-brain coherence index.

The PLI was then computed as in (2), as an index of the asymmetry of the distribution of signal instantaneous phase differences (Δφ), which were determined using the Hilbert transformation.

(2) $$PLI=|<(sign(\Delta \varphi \left(t_{k}\right))>|$$

The PLI values range between 0 and 1, where zero indicates a symmetric distribution, meaning either no coupling or coupling with a phase difference centered around 0 (mod π). The value of PLI increases as the strength of non-zero phase locking increases; thus, a PLI of 1 indicates perfect phase locking. For each epoch, a squared 52 × 52 PLI adjacency matrix was constructed. From this matrix, the global value (Mean PLI) was obtained by averaging those related to single pairs of channels. 

Finally, we applied graph theory building a minimum spanning tree (MST), which is defined as an acyclic (i.e., with no loops) connected graph, with N nodes and m = N − 1 links, where the sum of the weights, assigned to the links to represent the strength of connections of nodes, is minimized [45]. 

**Figure 3:**
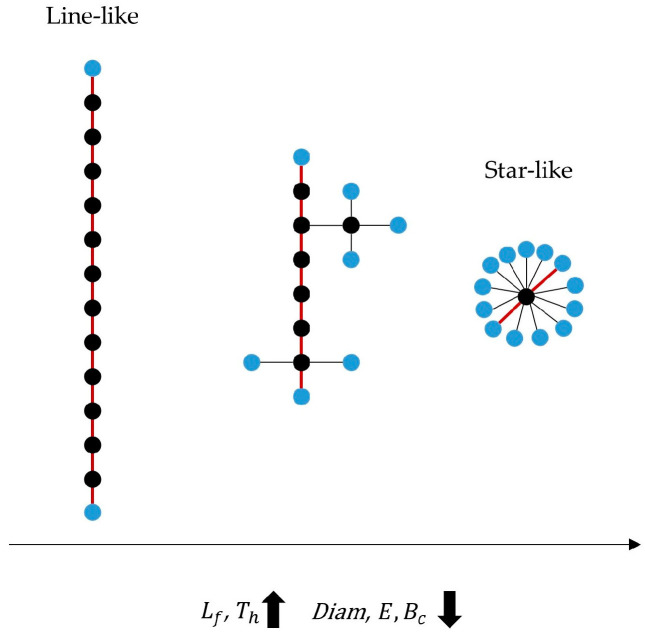
Schematic illustration of different tree topologies. For each configuration, leaf nodes are highlighted in blue; bold red lines refers to the *diameter* of the tree. The trend of the main parameters is shown as the type of configuration changes. Abbreviations: *L_f_* = *leaf fraction*, *T_h_* = *tree hierarchy*, *Diam* = *diameter*, *E* = *eccentricity*, *B_c_* = *betweenness centrality*.

The MST was derived by applying Kruskal’s algorithm [46], where the nodes of the graph correspond to the EEG channels, and the weights of the connections were computed based on the PLI matrix. The choice of using the PLI to compute the MST was based on the fact that, as stated in the Section 1, the PLI is more robust than the MSC, since it is less affected by the problem of volume conduction, which is higher in infant data [10]. In terms of tree topology, two extreme shapes exist (Figure 3). The first one is a line-like configuration, where all the nodes are connected to two other nodes, with the exception of the two nodes at either end. The opposite configuration is the star-like, where a central node is connected to all other nodes with one link. Between these two extreme topologies, many different types of tree configurations are possible [45]. 

The metrics, derived from the MST graph as explained in [45], characterizing the network topology are presented in Table 2 and briefly described below.

The degree of a node is defined as the number of connections with neighbor nodes. A node with degree of 1 is called leaf, and the number of such nodes over the total number of nodes is referred as the *leaf fraction* ($L_{f}$) of the tree. Thus, $L_{f}$ is bounded between 2/N, when the tree has a line-like topology, and (N − 1)/N for a star-like configuration. The level of information integration within the network could be assessed analyzing the *leaf fraction*, since a lower value indicates a less centralized network topology [47].

The *diameter* of a tree is the longest distance (expressed in number of edges) between any two nodes of the tree. It can be thought of as an index of efficiency of communication within the network. More in detail, a small diameter allows a more efficient information transmission among nodes [41].

The *eccentricity* of a node is defined as the number of edges of the longest path from this node to any other node. Lower values refer to nodes that are more central.

The *betweenness centrality* ($B_{c}$) of a single node was measured as the fraction of shortest paths between any pair of nodes running through that considered node. This way, the importance and centrality of each node within the network were assessed. For instance, in a star-like tree, the central node has a $B_{c}$ = 1 while the leaf-nodes have a $B_{c}$ of 0. 

Network performance and hierarchical topology of the tree was assessed by means of the *tree hierarchy* ($T_{h})$ metric, defined as in (3):

(3) $$T_{h}=\frac{L_{f}}{2mB_{max}}$$

where *m* is the total number of edges and, $B_{max}$ is the highest *betweenness centrality* of the tree. 

$T_{h}$ ranges between 0 and 1: if the leaf number is equal to 2 (i.e., line-like topology), then $T_{h}$ approaches 0, whereas with a leaf number equal to m (i.e., star-like configuration), $T_{h}$ approaches 0.5. This parameter quantifies the trade-off between large-scale integration of the tree and the overload of central nodes. Specifically, a combination of short distances between nodes and prevention of overload of any node ensures an optimal network organization.

Additionally, the *degree correlation* was quantified by computing the Pearson correlation coefficient between the degree of a node and the degree of its neighboring vertices to which it is connected. This metric can be considered an index of the robustness of a network. Biological networks are often characterized by a dissortative nature [48], (i.e., negative *degree correlation*). This means that nodes with higher degrees tend to connect to nodes with lower degrees and vice versa so that high-degree nodes are less connected to each other. As a result, the failure of a high-degree node in a dissortative network would have a significant impact on the network’s connectivity. 

Finally, the *kappa* metric was computed as in (4) to estimate the degree divergence, which is a measure of the broadness and heterogeneity of the degree distribution. It relates to the spread of information across the tree, since a low value of *kappa* indicates a low number of highly connected nodes [47].

(4) $$K=\frac{<k^{2}>}{<k>}$$

For those metrics that were calculated for each node, the mean value was then computed to globally characterize the tree.

Trends in MST parameters could then be interpreted to identify network modifications in terms of a shift toward a more line-like (i.e., less integrated) or a more star-like (i.e., more integrated) configuration.

Feature extraction was performed for each epoch and averaged across each subject in the delta (2–4 Hz), theta (4–6 Hz), low alpha (6–9 Hz), high alpha (9–13 Hz), beta (13–30 Hz) and gamma (30–45 Hz) frequency bands separately, with MATLAB R2023a.

Frequency band limits were set according to [49,50,51,52,53].

**Figure 4:**
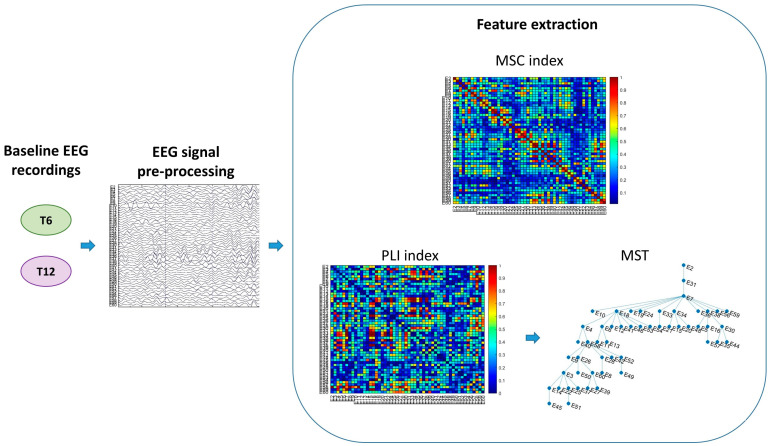
Schematic illustration of signal processing.

The signal processing pipeline is illustrated schematically in Figure 4.

### 2.4. Cognitive and Language Outcome Assessment at 24 Months of Age

The Bayley Scales of Infant and Toddler Development-3rd edition [37] was administered to the recruited infants at 24 months year of age (T24), to assess their language and cognitive development. In the present study, the raw cognitive score (hereafter: RC) was used as the measure of cognitive developmental status, while receptive and expressive language scores were adopted as measures of the language (i.e., vocabulary and morpho-syntactic) developmental status. To globally assess the language outcome, a composite language metric (hereafter: RLC) was computed as the mean value of the raw scores of the receptive and expressive scale sections and used for further analysis.

Some attrition was present at the 2-year follow-up Bayley scores data collection. The RC metric was collected for the 48.5% of the infants recruited at T6 and the 55.7% of the infants of the T12 group, whereas the RLC score was stored for the 50% of the participants of the T6 group and the 54.1% for the T12 group. The assumption of data missing completely at random was tested with the Little’s Missing Completely at Random (MCAR) test, performed with SPSS statistics (version 21, Chicago, IL, USA). These statistics showed that missing data in the measures collected at 24 months were random (follow-up of the 6-month-old measures: χ^2^(29) = 20.31, *p* = 0.883; follow-up of the 12-month-old measures: χ^2^(19) = 19.23, *p* = 0.442).

### 2.5. Statistical Analysis

Data normality was checked with the Kolmogorov–Smirnov test, and the appropriate statistic was applied. Specifically, to investigate how FC varies between 6 and 12 months of age, also accounting for sex-related effects (main aim of the work), a generalized linear mixed-effect model (GLMM) was used to accommodate fixed and random effects within the 6-month-old infants and their 12-month-old peers, testing for significant differences in the computed FC metrics, while adjusting for missing data and accounting for the non-independency of data arising from participants. The time point (T6 or T12) of the data acquisition and the sex of subjects were used as fixed effects; while the interaction between these two effects was also investigated.

For all statistical tests, a *p* value correction for multiple comparison was performed applying the false discovery rate (FDR) method [54]. Significance level was then set at adjusted *p* < 0.05.

Furthermore, in order to fulfill our secondary aim, the correlation between FC metrics in the gamma frequency band and the Bayley scale scores was explored by means of the Spearman’s correlation coefficient. A 1000-step bootstrap technique was applied [55] to obtain more robust results. Only confidence intervals (CIs) that did not contain the value 0 were considered for significant correlation effects. Due to the exploratory nature of these correlational analyses, we did not adjust significance levels for multiple testing, in order to avoid type-II errors and not to miss potentially relevant associations [56].

All statistical analysis was performed with SPSS statistics (version 21, Chicago, IL, USA).

## 3. Results

Table 3 reports the resulting *p* values of the GLMM and the adjusted *p* values for all the computed features in all the frequencies, for the time point and sex fixed effects. No significant interaction was found between the two considered effects. Adjusted *p* values were considered for further analyses and considerations. 

**Figure 5:**
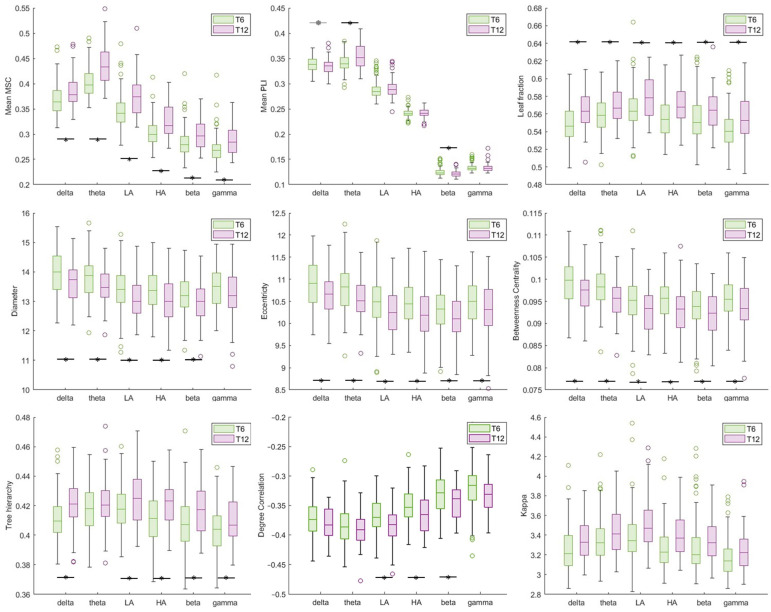
Boxplots reporting time point-related effect (T6 vs. T12) on FC features. Asterisks represent statistically significant differences (adjusted *p* < 0.05); while gray asterisks represent trends (adjusted *p* < 0.07).

Additionally, time point-related differences are displayed in Figure 5. More in detail, the majority of the FC features showed a significant variation between the T6 and T12 time points in all the frequency bands, suggesting an increased functional connectivity at 12 months of age with respect to 6 months: the Mean MSC and Mean PLI features were significantly higher in the T12 group; the MST analysis highlighted increased *leaf fraction, tree hierarchy, degree correlation* and *kappa* features and decreased *diameter*, *betweenness centrality* and *eccentricity* parameters in the T12 group. 

As for sex effect, males and females displayed significant differences only in the *tree hierarchy* parameter in the high alpha frequency band, while a variation trend in the gamma frequency for the Mean MSC and *leaf fraction* features was also observed. All these three parameters suggested higher functional connectivity in the female group with respect to males. 

**Figure 6:**
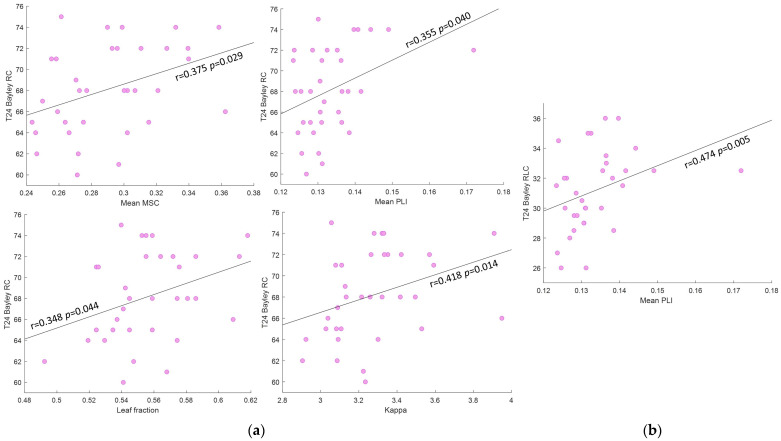
Significant correlations between FC metrics in the gamma frequency band at T12 and Bayley scale scores. (**a**) Correlations between FC metrics and the RC scores; (**b**) Correlations between FC metrics and the RLC scores. Abbreviations: RC = raw cognitive score, RLC = raw language composite score, MSC = magnitude squared coherence, PLI = phase lag index.

Results from the correlational analyses between FC parameters in the gamma frequency band and the cognitive and language Bayley scale scores are displayed in Table 4 for T6-features and in Table 5 for T12-features. Significant correlations were found only between metrics extracted at 12 months and mainly cognitive performances. In particular, the Mean MSC, the Mean PLI, and the *kappa* features showed positive correlations with RC scores, demonstrating an increased cognitive level as functional connectivity becomes stronger. A similar correlation trend was obtained with the *leaf fraction*, which showed a *p* value < 0.05 but a confidence interval that marginally contains the 0 value (Table 5). Moreover, the Mean PLI presented also a significant positive correlation with the composite language score. Figure 6 illustrates significant correlations for the T12-FC features.

## 4. Discussion

The present study aimed to assess network developmental changes in infants in the second half of their first year of life, which is a crucial period for brain development and the acquisition of cognitive functions. For this purpose, baseline EEG data of 146 typically developing infants were collected at 6 and 12 months of age. Brain maturation from 6 to 12 months was measured by means of FC features for multiple frequency bands between all pairs of channels. As reported in [10], the problem of volume conduction is higher during EEG recording in infants. For this reason, we decided to include in our study other FC metrics, besides the most common used magnitude-squared coherence (i.e., phase lag index and graph analysis) that could overcome this problem so as to obtain more robust results.

The results obtained from the GLMM, performed considering the time point and sex as fixed effects, highlighted an increased functional connectivity, for all frequency bands, in 12-month-old infants with respect to their 6-month-old peers. Specifically, statistically higher Mean MSC and Mean PLI were found in the T12 group, suggesting greater coherence and coupling between channels, thus among the brain network, with development. Additionally, the increase in the *leaf fraction* and *tree hierarchy* MST features and coherently the decrease in the *diameter*, *eccentricity* and *betweenness centrality* metrics of the graph are representative of a development-related shift from a line-like to a more star-like network configuration, characterized by a better organization, efficiency of communication and integration of the information between nodes. These findings are confirmed also by literature evidence that demonstrates through fMRI study an improved functionality of the brain during the first year of life [15]. Moreover, research about structural changes seem, to corroborate these results, reporting a greater integration and efficiency of neural networks during the first years of life [14].

Sex differences in brain connectivity were also investigated by means of the GLMM, finding higher FC in females with respect to males, only in a small number of features (i.e., Mean MSC, *leaf fraction*, *tree hierarchy*) and mainly in the gamma (at trend level) and high alpha frequency bands, despite the evidence of the influence of sex on differences in brain function maturation [20]. Although these preliminary results are overall in line with the literature findings [21,22], they will not be further discussed given their limited significance. Further studies are necessary to explore gender differences on FC, especially during the early stages of brain development. 

Furthermore, we performed an exploratory investigation of the relation between functional connectivity in the gamma frequency and cognitive and language development, since activities in gamma frequencies are proven to be associated with cognitive processing and abilities and language development (e.g., [32,34,57]). No significant correlation was found with features extracted at 6 months of age. Conversely, the cognitive performance assessed at 24 months of age significantly correlated with the Mean MSC, the Mean PLI, the *leaf fraction* and *kappa* FC metrics, all extracted from EEG data at 12 months. Moreover, a significant correlation was found between the composite language scores and the Mean PLI feature at T12. The lack of significant correlation with T6-FC metrics can be explained by hypothesizing that, at this age, the brain network is not fully and well organized [15]. Consequently, differences in cognitive skills at 24 months of age could not yet be reflected in the brain functional connectivity at 6 months. On the other hand, at 12 months of age, FC has become stronger, thus an increase in the coherence, stronger coupling (i.e., increasing PLI), higher integration of information within the network (i.e., higher *leaf fraction*) and increasing of number of highly connected nodes (i.e., higher *kappa*) could be reflected in enhanced cognitive performance. These results suggest that functional connectivity of brain networks at 12 months of age may be a marker of later cognitive development. 

Nevertheless, it is important to acknowledge some limitations of the present study. First, we recognize that the procedure of collecting baseline EEG data in infants, which foresees the use of an entertainment activity, such as blowing bubbles, might introduce some confounds. This practice is widely accepted in infant studies [19] and allows to record data in a reproducible situation. Moreover, EEG signals were pre-processed in order to obtain as clean data as possible, removing the data in which large movement artifacts were present, thus extracting the more reliable outcomes.

Secondly, although our study represents one of the first attempts to investigate EEG FC at different stages of early brain maturation, it was limited to a period between 6 and 12 months of age; a longitudinal study with multiple time points would allow to delineate eventual nonlinear growth trends, ensuring a more comprehensive evaluation during infancy and also childhood. It is also worth noticing that only 47 infants whose data were collected at T6 performed baseline EEG also at T12. Despite missing values, the sample size can be considered adequate for the purpose of the study.

In addition, the longitudinal measures of cognitive and language skills were available only for 74 subjects out of the 146 recruited for the study; however, the sample size on which the analyses were conducted remains statistically adequate, and the Little’s MCAR test showed that missing data were random. 

Finally, the focus of our study is brain functional connectivity analysis and its link to cognitive outcomes, without considering possible effective connectivity-related outcomes. Further developments should thus combine structural, functional and effective connectivity in a longitudinal study to globally assess brain maturation throughout infancy and childhood so as to better understand the intrinsic relationship and constraints between brain functionality and microstructural properties and how they can change together over time [58], while also accounting for causal interactions between neural elements. When dealing with functional connectivity analysis, future developments should also consider using different FC metrics, such as the weighted PLI (wPLI) or the phase locking value (PLV), which seem to be more reliable [9,59], thus supporting our finding.

Finally, future studies may also focus on a deeper investigation on sex-related differences in FC and corroborate our preliminary findings. Lastly, future works should aim to confirm the possibility of exploiting functional connectivity measures, extracted from EEG data recorded from 12 months of age, to predict cognitive developmental status at 24 months or at older ages, performing analyses with a larger sample size.

To conclude, the present study contributes to the understanding of functional brain network evolution during the first year of life on typically developing infants. To obtain this aim, we proposed the use of FC derived from baseline EEG data, rather than the more common and widely used fMRI techniques. Our results are in line with the fMRI literature thus supporting the use of EEG technique to investigate brain FC in developmental populations, as it ensures a superior temporal resolution and higher suitability for very young age groups [13]. This study also represents one of the first attempts to assess whether functional maturation at 6 or 12 months of age can be reflected into differences in cognitive performance at 24 months, thus identifying possible functional markers of later cognition.

## Figures and Tables

**Table 1:** Subjects’ sociodemographic and individual factors, Mean (SD).

Age (days)	T6: 197.6 (13.99) T12: 377.16 (11.98)
Sex	70 M 76 F
Gestational age (weeks)	39.37 (1.33)
Birth weight (g)	3279.85 (401.9)
Cognitive Score at T6 ^a^	107.04 (8.75)
Socio-economic status at T6 ^b^	66.34 (16.18)

^a^ Bayley Cognitive composite score (26) or Griffiths developmental quotient (27). ^b^ SES was scored according to the Hollingshead 9-point scale, whereby a score ranging from 10 to 90 was assigned to each parental job, and the higher of the two scores was used when both parents were employed [36].

**Table 2:** Minimum spanning tree features description.

MST Features	Description
Leaf fraction	Number of nodes with a single connection
Diameter	Longest distance between any two nodes
Eccentricity	Longest distance between a reference node and any other node
Betweenness Centrality	Fraction of all shortest paths that pass through a particular node
Tree hierarchy	Hierarchical metric of the network organization
Degree Correlation	Correlation between the degree of a node and the degree of the nodes to which it is connected
Kappa	Degree divergence

**Table 3:** Generalized linear mixed model outcome. Both *p* values and adjusted *p* values are reported for the time point and sex fixed effects. Bold text with one asterisk represents result at trend level (0.05 < adjusted *p* < 0.07); significance is highlighted with double asterisk (adjusted *p* < 0.05), while three asterisks are present for adjusted *p* < 0.01.

		Time Point Effect			Sex Effect		
FC Feature	Frequency Band	*F*	*p* Value	adj *p* Value	*F*	*p* Value	adj *p* Value
Mean MSC	Delta	14.243	<0.001	<0.001 ***	0.0596	0.441	0.529
	Theta	53.884	<0.001	<0.001 ***	0.200	0.655	0.655
	Low Alpha	31.638	<0.001	<0.001 ***	0.817	0.367	0.529
	High Alpha	26.201	<0.001	<0.001 ***	2.723	0.101	0.228
	Beta	16.135	<0.001	<0.001 ***	2.519	0.114	0.228
	Gamma	18.833	<0.001	<0.001 ***	6.532	0.011	0.066 *
Mean PLI	Delta	5.012	0.026	0.052 *	0.936	0.335	0.503
	Theta	27.958	<0.001	<0.001 ***	2.174	0.142	0.426
	Low Alpha	1.014	0.315	0.473	1.316	0.253	0.503
	High Alpha	0.164	0.686	0.823	0.349	0.555	0.555
	Beta	8.923	0.003	0.009 ***	0.42	0.518	0.555
	Gamma	0.013	0.909	0.909	3.761	0.054	0.324
Leaf fraction	Delta	19.385	<0.001	<0.001 ***	0.094	0.760	0.820
	Theta	13.929	<0.001	<0.001 ***	3.949	0.048	0.096
	Low Alpha	14.827	<0.001	<0.001 ***	0.052	0.820	0.820
	High Alpha	24.281	<0.001	<0.001 ***	5.124	0.025	0.075
	Beta	10.381	0.001	0.0012 ***	2.015	0.157	0.236
	Gamma	10.292	0.002	0.002 ***	6.676	0.011	0.066 *
Diameter	Delta	10.359	0.002	0.004 ***	0.112	0.739	0.887
	Theta	11.128	0.001	0.004 ***	0.011	0.918	0.918
	Low Alpha	7.635	0.006	0.009 ***	0.428	0.514	0.887
	High Alpha	9.957	0.002	0.004 ***	1.398	0.239	0.726
	Beta	4.923	0.028	0.034 **	0.205	0.651	0.887
	Gamma	3.125	0.079	0.079	1.375	0.242	0.726
Eccentricity	Delta	9.427	0.002	0.006 ***	0.213	0.645	0.821
	Theta	11.483	0.001	0.006 ***	0.002	0.967	0.967
	Low Alpha	7.567	0.007	0.011 **	0.166	0.684	0.821
	High Alpha	8.579	0.004	0.008 ***	0.508	0.477	0.821
	Beta	4.117	0.044	0.046 **	0.207	0.650	0.821
	Gamma	4.051	0.046	0.046 **	0.275	0.275	0.821
Betweenness Centrality	Delta	10.036	0.002	0.004 ***	0.185	0.668	0.992
	Theta	15.703	<0.001	<0.001 ***	0	0.992	0.992
	Low Alpha	10.087	0.002	0.004 ***	0.007	0.931	0.992
	High Alpha	9.317	0.003	0.0045 ***	0.587	0.445	0.992
	Beta	4.207	0.042	0.042 **	0.219	0.640	0.992
	Gamma	4.482	0.036	0.042 **	2.074	0.152	0.912
Tree hierarchy	Delta	20.436	<0.001	<0.001 ***	0.012	0.913	0.913
	Theta	3.113	0.079	0.079	3.636	0.058	0.174
	Low Alpha	9.576	0.002	0.004 ***	0.108	0.743	0.892
	High Alpha	18.796	<0.001	<0.001 ***	9.19	0.003	0.018 **
	Beta	8.192	0.005	0.0075 ***	2.015	0.157	0.236
	Gamma	5.959	0.016	0.019 **	2.949	0.088	0.176
Degree Correlation	Delta	3.864	0.051	0.0765	1.088	0.298	0.439
	Theta	1.094	0.297	0.297	2.029	0.156	0.312
	Low Alpha	14.582	<0.001	<0.001 ***	0.306	0.581	0.581
	High Alpha	7.81	0.006	0.016 **	0.821	0.366	0.439
	Beta	7.081	0.008	**0.** 016 **	4.529	0.035	0.141
	Gamma	3.173	0.076	0.0912	4.009	0.047	0.141
Kappa	Delta	8.977	0.003	0.006 ***	0.344	0.558	0.665
	Theta	8.256	0.005	0.006 ***	4.638	0.033	0.099
	Low Alpha	13.531	<0.001	<0.001 ***	0.188	0.665	0.665
	High Alpha	20.229	<0.001	<0.001 ***	2.758	0.098	0.196
	Beta	3.47	0.064	0.064 *	1.509	0.221	0.332
	Gamma	8.366	0.004	0.006 ***	5.544	0.020	0.099

**Table 4:** Correlation matrix of T6-FC metrics in the gamma frequency band and Bayley scale scores. Abbreviations: CI = Confidence interval, RC = raw cognitive score, RLC = raw composite language score; MSC = magnitude squared coherence, PLI = phase lag index, Diam = diameter, E = eccentricity, B_c_ = betweenness centrality, T_h_ = tree hierarchy.

		Mean MSC	Mean PLI	Leaf Fraction	Diam	E	B_c_	T_h_	Degree Corr.	Kappa
T24 Bayley RC	r	−0.033	0.118	−0.031	0.151	0.138	0.106	−0.077	0.072	−0.040
	*p* value	0.796	0.354	0.809	0.233	0.279	0.406	0.543	0.570	0.754
	CI	[−0.32, 0.25]	[−0.13, 0.36]	[−0.3, 0.25]	[−0.1, 0.41]	[−0.12, 0.38]	[−0.16, 0.37]	[−0.33, 0.19]	[−0.22, 0.36]	[−0.33, 0.24]
T24 Bayley RLC	r	−0.116	0.111	0.075	−0.141	−0.183	−0.203	−0.076	0.080	0.106
	*p* value	0.352	0.374	0.547	0.259	0.141	0.102	0.544	0.522	0.397
	CI	[−0.38, 0.15]	[−0.14, 0.36]	[−0.2, 0.34]	[−0.39, 0.13]	[−0.43, 0.08]	[−0.45, 0.06]	[−0.34, 0.19]	[−0.18, 0.33]	[−0.14, 0.36]

**Table 5:** Correlation matrix of T12-FC metrics in the gamma frequency band and Bayley scale scores. Significant correlations are highlighted by bold text and asterisks. Abbreviations: CI = Confidence interval, RC = raw cognitive score, RLC = raw composite language score; MSC = magnitude squared coherence, PLI = phase lag index, Diam = diameter, E = eccentricity, B_c_ = betweenness centrality, T_h_ = tree hierarchy.

		Mean MSC	Mean PLI	Leaf Fraction	Diam	E	B_c_	T_h_	Degree Corr.	Kappa
T24 Bayley RC	r	**0.375**	**0.355**	**0.348**	−0.104	−0.131	−0.227	0.096	−0.076	**0.418**
	*p* value	0.029 *	0.040 *	0.044 *	0.560	0.459	0.197	0.589	0.670	0.014 *
	CI	**[0.04, 0.63]**	**[0.01, 0.64]**	**[−0.001, 0.61]**	[−0.44, 0.29]	[−0.46, 0.27]	[−0.53, 0.17]	[−0.28, 0.45]	[−0.39, 0.26]	**[0.06, 0.67]**
T24 Bayley RLC	r	0.114	**0.474**	0.046	0.247	0.207	0.051	−0.087	0.104	0.115
	*p* value	0.528	0.005 *	0.799	0.166	0.248	0.780	0.629	0.564	0.525
	CI	[−0.24, 0.46]	**[0.13, 0.71]**	[−0.32, 0.39]	[−0.12, 0.56]	[−0.17, 0.53]	[−0.34, 0.4]	[−0.46, 0.27]	[−0.23, 0.44]	[−0.21, 0.43]

## Data Availability

The original data presented in the study, including subjects’ sociodemographic and individual factors and all individual FC metrics and Bayley test raw scores used to perform the statistical analysis, are deposited on Zenodo and openly available as of the date of publication (Zenodo: https://doi.org/10.5281/zenodo.11621601, accessed on 17 June 2024).
